# Expression Analysis Reveals the Association of Several Genes with Pupal Diapause in *Bactrocera minax* (Diptera: Tephritidae)

**DOI:** 10.3390/insects10060169

**Published:** 2019-06-13

**Authors:** Jia Wang, Huan Fan, Pan Wang, Ying-Hong Liu

**Affiliations:** College of Plant Protection, Southwest University, Chongqing 400716, China; fanhcc@163.com (H.F.); wpjyoio@163.com (P.W.); yhliu@swu.edu.cn (Y.-H.L.)

**Keywords:** *Bactrocera minax*, pupal diapause, gene expression, 20-hydroxyecdysone

## Abstract

The Chinese citrus fly, *Bactrocera minax*, is a devastating pest of citrus, which enters the obligatory diapause in overwintering pupae to resist harsh environmental conditions. However, little is known about the molecular mechanisms underlying pupal diapause. The previous transcriptomic analysis revealed that a large number of genes were regulated throughout the pupal stage. Of these genes, 12 and six ones that are remarkably up- and downregulated, respectively, specifically in intense diapause were manually screened out in present study. To validate the expression of these genes throughout the pupal stage, the quantitative real-time PCR (qRT-PCR) was conducted, and the genes displaying different expression patterns with those of previous study were excluded. Then, the expressions of remaining genes were compared between diapause-destined and non-diapause-destined pupae to reveal their association with diapause using qRT-PCR and semiquantitative PCR. Finally, five genes, *TTLL3B*, *Cyp6a9*, *MSTA*, *Fru*, and *UC2*, and two genes, *KSPI* and *LYZ1*, were demonstrated to be positively and negatively associated with diapause, respectively. These findings provide a solid foundation for the further investigation of molecular mechanisms underlying *B. minax* pupal diapause.

## 1. Introduction

The Chinese citrus fly, *Bactrocera minax* (Enderlein) (Diptera: Tephritidae), an oligophagous pest of citrus fruits, has been considered as a devastating pest of citrus plants in the temperate areas of Asia [1,2]. Usually, the female adults lay eggs into the citrus fruit where the larvae hatch and feed, causing the fruits to ripen prematurely and drop down. Subsequently, the mature larvae drill out of the fallen fruits and burrow into soil for pupation [2]. Recently, the outbreaks of *B. minax* in several provinces of China have caused serious economic losses to the citrus industry [2,3]. Therefore, this pest has aroused great concerns in citrus-growing regions in China and the research on this pest has been widely conducted [4,5,6,7,8,9,10,11].

Diapause is a genetically programmed developmental arrest, accompanied by suppressed metabolism and enhanced stress tolerance [12,13], in response to environmental stimuli or internal cues to resist adverse environmental conditions in unfavorable season [14]. Many univoltine insects enter obligatory diapause at a specific developmental stage in each generation but require no token stimuli for diapause induction and preparation [12]. Likewise, the univoltine *B. minax* also enters the pupal diapause to overwinter. The overwintering pupal stage lasts for 160–170 d, synchronizing the adult emergence with citrus fruit bearing [15]. Therefore, understanding the molecular mechanisms of pupal diapause of *B. minax* will contribute to elucidating the inherent mechanisms underlying pupal development and adaptation to harsh environment. In the past decade, several relevant studies on pupal diapause of *B. minax* had been carried out in China. For example, the previous studies have demonstrated that the chilling in diapause is critical for pupal survival and adult emergence [10], and the 20E application was able to terminate pupal diapause and significantly accelerate adult emergence [7,16]. Moreover, the respiratory rate throughout the pupal stages was measured, and the fitted respiratory rate trajectory indicated that *B. minax* gradually entered intense diapause approximately 40 d after pupation, and the intense diapause lasted for two months (40–100 d after pupation) [17]. Although substantial progress has been made, the understanding of mechanisms underlying pupal diapause of *B. minax* is still limited.

Mining the diapause-associated genes could be an effective way in an effort to reveal the mechanisms underlying diapause. A number of studies have been conducted in this endeavor and many diapause-associated genes have been discovered in insects [18,19,20,21]. Recently, the next-generation sequencing technology has widely been used to study the mechanisms underlying a lot of biological processes in organisms by revealing the gene expression profiles in high-throughput and large-scale manners [22,23,24]. Given the importance of insect diapause, the underlying mechanisms have been studied using next-generation sequencing technology in many insects [25,26,27]. In our previous study, the transcriptome of *B. minax* was obtained using next-generation sequencing technology, based on which the gene expression profiles throughout pupal stage were compared, and a large number of genes were found to be regulated specifically in diapause [17]. In order to reveal the physiological variation in diapause, genes regulated most remarkably during diapause were manually screened out in this study, according to the reads per kb of exon model per million mapped reads (RPKM) values obtained previously [17]. Then, the quantitative real-time PCR (qRT-PCR) was conducted to verify the expression patterns of these genes throughout the pupal stage. Those genes which displayed inconsistent expression patterns between qRT-PCR and transcriptomic analysis were excluded. To further verify the association of remaining genes with diapause, the qRT-PCR and semiquantitative PCR were performed to compare the gene expression levels between diapause-destined and non-diapause-destined pupae. The findings of this study laid basis for further investigations on the mechanisms underlying *B. minax* diapause.

## 2. Materials and Methods

### 2.1. Insect Rearing and Sample Collection

Fallen oranges infested with maggots were taken back to the lab from an orchard in Wulong County, Chongqing Municipality, China. Third-instar larvae were collected from oranges and placed over sand in plastic dishes to pupate. All pupae were reared at 18 ± 2 °C, 70 ± 10% relative humidity, and photoperiod of 14 L:10 D. Twenty pupae were collected and stored in liquid nitrogen every 10 days until adult emergence for subsequent gene expression analysis.

### 2.2. Acquisition of Non-Diapause-Destined Pupae and Sample Collection

Non-diapause-destined pupae were acquired by injecting 20E into newly-formed pupae. The 20E (Sigma, St. Louis, MO, USA) was dissolved in 10% ethanol to concentration of 1 μg/μL. Each newly-formed pupa with the same size (≈9 mm) was injected with 1 μL 20E solution. Then all pupae were reared in plastic dishes under the same condition described above. Twenty pupae were collected and stored in liquid nitrogen every 10 days for subsequent gene expression analysis.

### 2.3. RNA Isolation and First-Strand cDNA Synthesis

Total RNA was isolated from whole body of pupa using TRIzol Reagent (Life Technologies, Carlsbad, CA, USA) according to manufacturer’s protocols. The integrity of RNA was detected by 1% agarose gel electrophoresis. Reverse transcription of 500 ng total RNA into the first-strand cDNA was performed using a PrimeScript^TM^ RT Master Mix (Perfect Real Time) Kit (Takara, Shiga, Japan). Three biological replicates were set for each time point.

### 2.4. Selection of Putative Diapause-Associated Genes

The previous transcriptomic study discovered significantly differentially expressed genes (DEGs) throughout pupal stage according to the RPKM values. The cluster analysis indicated that 1434 and 848 DEGs were up- and downregulated, respectively, specifically in diapause (40–100 d after pupation) [17]. In this study, the top hit DEGs with stable expression among biological replicates were manually selected as putative highly diapause-associated genes. These genes were annotated by alignment to NCBI Nr database using BLASTx (http://www.ncbi.nlm.nih.gov/) (Table 1).

### 2.5. Identification of Highly Diapause-Associated Genes

The expression profiles of 18 selected genes throughout pupal stage were analyzed by qRT-PCR. The genes displaying different expression patterns between qRT-PCR analysis and previous transcriptomic study were excluded. Then, the expression levels of remaining genes were compared between diapause-destined and non-diapause-destined pupae, using qRT-PCR and semiquantitative PCR, to determine the highly diapause-associated genes. The specific primers for 18 putative genes were designed (Table S1), and *UBQ* was used as the reference gene [28]. The qRT-PCR was performed with a CFX96^TM^ Real-Time PCR Detection System thermal cycler (BIO-RAD, Hercules, CA, USA) in a reaction volume of 10 μL, including 4 μL SYBR^®^ Premix Ex Taq II (Takara), 1 μL cDNA (50 ng/μL), and 0.5 μL forward and reverse primers (10 μM). Relative expression level was calculated according to the 2^−ΔΔCt^ method. Three technique replicates were set for each sample. For semiquantitative PCR, the reaction volume was 25 µL, including 2 µL 10× PCR reaction buffer, 2.5 μL dNTP Mix (10 mM), 2.5 μL MgCl_2_ (25 mM), 0.25 μL Taq polymerase (2.5 U), 1 μL forward primer (10 μM) and reverse primer (10 μM), 1 μL cDNA template, and 14.8 μL ddH_2_O. The PCR reaction was carried out under conditions: 94 °C for 5 min, 28–32 cycles of 94 °C for 30 s followed by 55–60 °C 30 s, 72 °C 30 s, 72 °C 5 min (Table S1). To confirm the identity of highly diapause-associated genes, phylogenetic analysis was performed by Molecular Evolutionary Genetics Analysis version 5 (MEGA 5, https://www.megasoftware.net/) with the neighbor-joining method on the basis of the amino acid sequences available in GenBank (http://www.ncbi.nlm.nih.gov/). The reliability of the branching was tested using bootstrap method (1000 replications).

### 2.6. Statistical Analysis

The relative expression level of each gene throughout pupal stages was compared using one-way analysis of variance (ANOVA) with Tukey’s test after verification of normality and homogeneity of variances. The relative expression level of each gene was compared between diapause-destined and non-diapause-destined pupae using independent *t*-test. All data analyses were performed with SPSS 22.0 software (SPSS Inc., Chicago, IL, USA).

## 3. Results

### 3.1. Expression Profiles of Selected Genes throughout Pupal Stage

**Figure 1:**
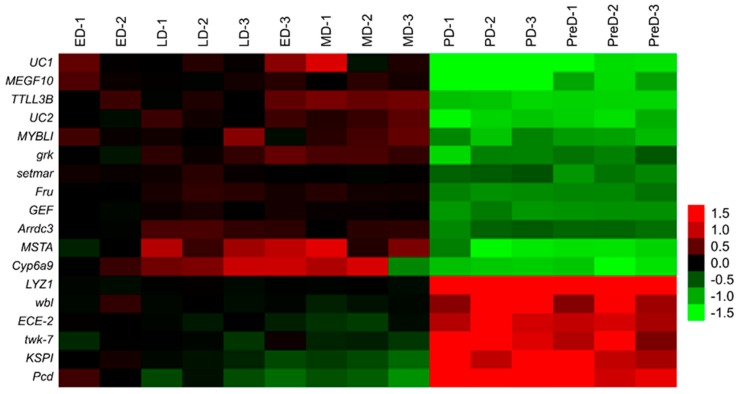
Heat map of expression levels of 18 putative diapause-associated genes throughout the pupal stage of *B. minax* according to the reads per kb of exon model per million mapped reads (RPKM) values. Red and green color gradients indicate the high and low expression levels, respectively. PreD, pre-diapause, 1 d after pupation; ED, early-diapause, 57 d after pupation; MD, middle-diapause, 75 d after pupation; LD, late-diapause, 95 d after pupation. PD, post-diapause, 135 d after pupation.

**Figure 2:**
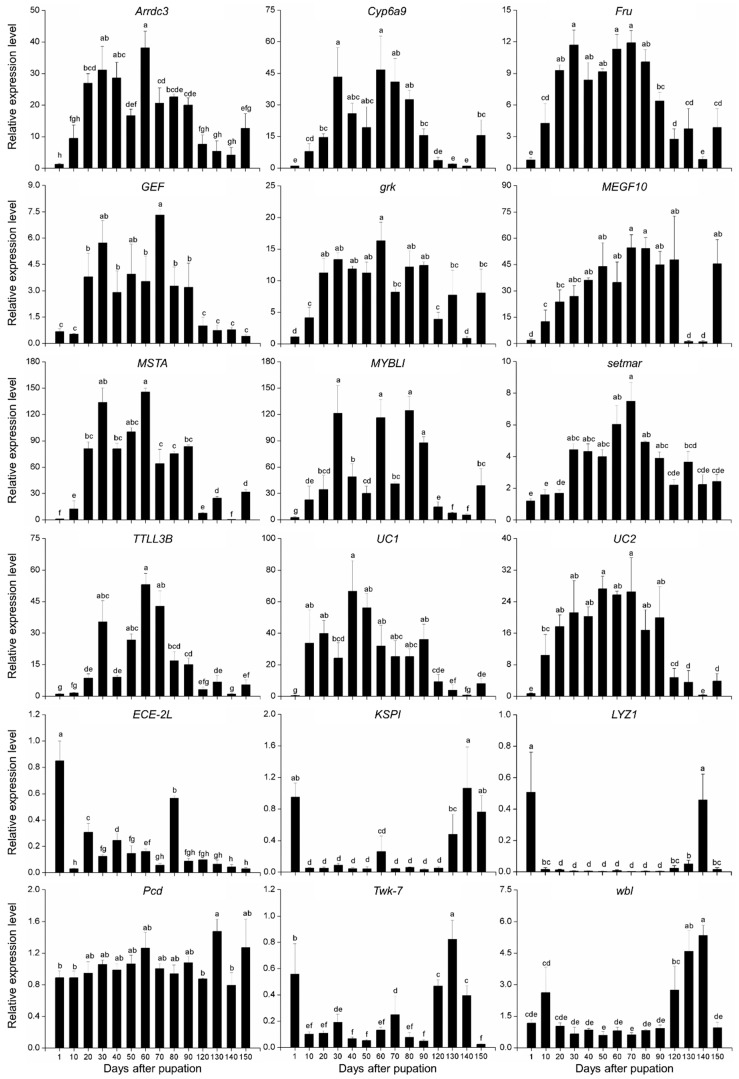
Relative expression levels of 18 putative diapause-associated genes throughout pupal stage of *B. minax*. For each gene, the relative expression level was calculated against a replicate of newly-formed pupae (1 d after pupation) according to the 2^−ΔΔCt^ method. Different letters indicate significant differences (*p* < 0.05).

A total of 12 upregulated and six downregulated genes during diapause were manually selected as putative diapause-associated genes according to the RPKM values (Figure 1). All eighteen genes were significantly regulated throughout the pupal stage. However, only 13 genes, *Arrdc3* (F_13,41_ = 12.931, *p* < 0.05), *Cyp6a9* (F_13,41_ = 37.071, *p* < 0.05), *Fru* (F_13,41_ = 19.433, *p* < 0.05), *GEF* (F_13,41_ = 14.4, *p* < 0.05), *KSPI* (F_13,41_ = 28.247, *p* < 0.05), *LYZ1* (F_13,41_ = 524.051, *p* < 0.05), *MSTA* (F_13,41_ = 205.801, *p* < 0.05), *MYBLI* (F_13,41_ = 18.417, *p* < 0.05), *setmar* (F_13,41_ = 11.244, *p* < 0.05), *TTLL3B* (F_13,41_ = 25.601, *p* < 0.05), *twk-7* (F_13,41_ = 66.57, *p* < 0.05), *UC1* (F_13,41_ = 19.150, *p* < 0.05)*,* and *UC2* (F_13,41_ = 23.198, *p* < 0.05), showed similar expression patterns with those of previous transcriptomic study. The remaining five genes, *ECE-2L* (F_13,41_ = 251.969, *p* < 0.05), *grk* (F_13,41_ = 24.785, *p* < 0.05), *MEGF10* (F_13,41_ = 7.35, *p* < 0.05), *Pcd* (F_13,41_ = 2.672, *p* < 0.05), and *wbl* (F_13,41_ = 17.428, *p* < 0.05), were excluded. The expression levels of *Arrdc3*, *Cyp6a9*, *Fru*, *GEF*, *MSTA*, *MYBLI*, *setmar*, *TTLL3B*, *UC1*, and *UC2* were upregulated along with intensification of diapause and downregulated after diapause termination, although the differences were not significant at certain time points. The expression levels of *KSPI*, *LYZ1*, and *twk-7* were remarkably suppressed 10 days after pupation and upregulated after diapause termination (Figure 2).

### 3.2. Comparison of Gene Expression Levels between Diapause-Destined and Non-Diapause-Destined Pupae

**Figure 3:**
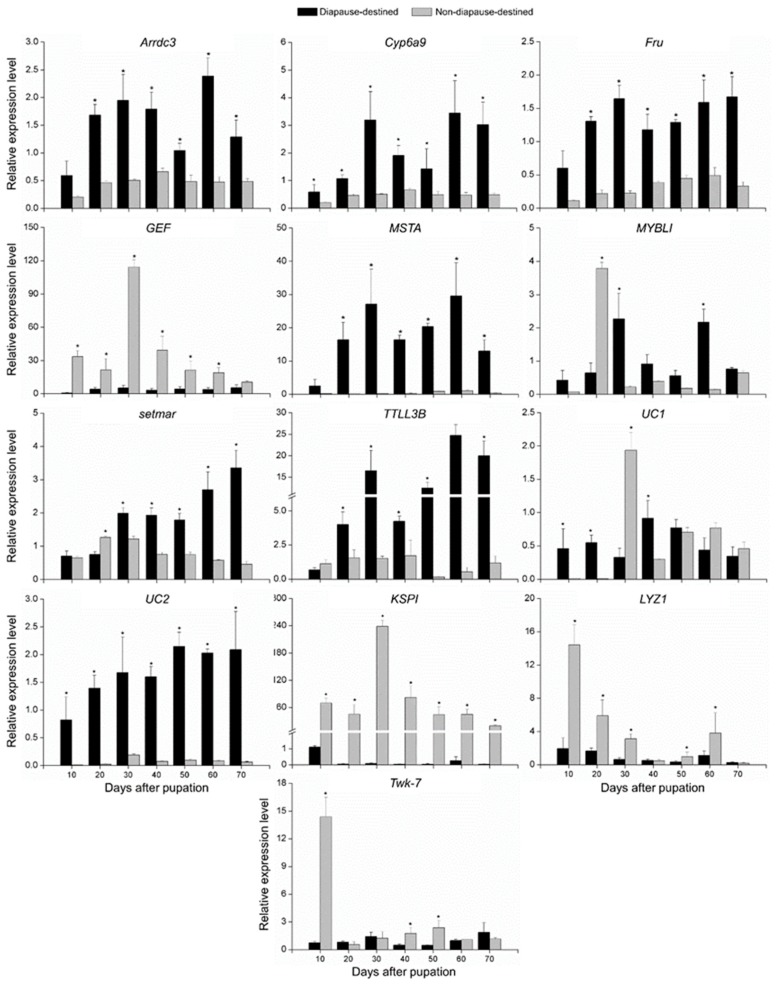
Relative gene expression levels between diapause-destined and non-diapause-destined *B. minax* pupae measured by qRT-PCR. Asterisks indicate significant differences (*p* < 0.05).

The newly-formed pupae were injected with 20E to acquire non-diapause-destined pupae. To validate the association of 13 genes mentioned above with diapause, their expression levels were compared between diapause-destined and non-diapause-destined pupae using qRT-PCR. Nine genes, *Arrdc3*, *Cyp6a9*, *Fru*, *KSPI*, *LYZ1*, *MSTA*, *setmar*, *TTLL3B*, and *UC2*, were found to be putatively diapause-associated. The expression of *Arrdc3*, *Cyp6a9*, *Fru*, *MSTA*, *setmar*, *TTLL3B*, and *UC2* were suppressed, while that of *KSPI* and *LYZ1* were activated in non-diapause-destined pupae (Figure 3). In addition, the *MYBLI*, *twk-7*, *UC1*, and *GEF* were not regulated as expected (Figure 3).

**Figure 4:**
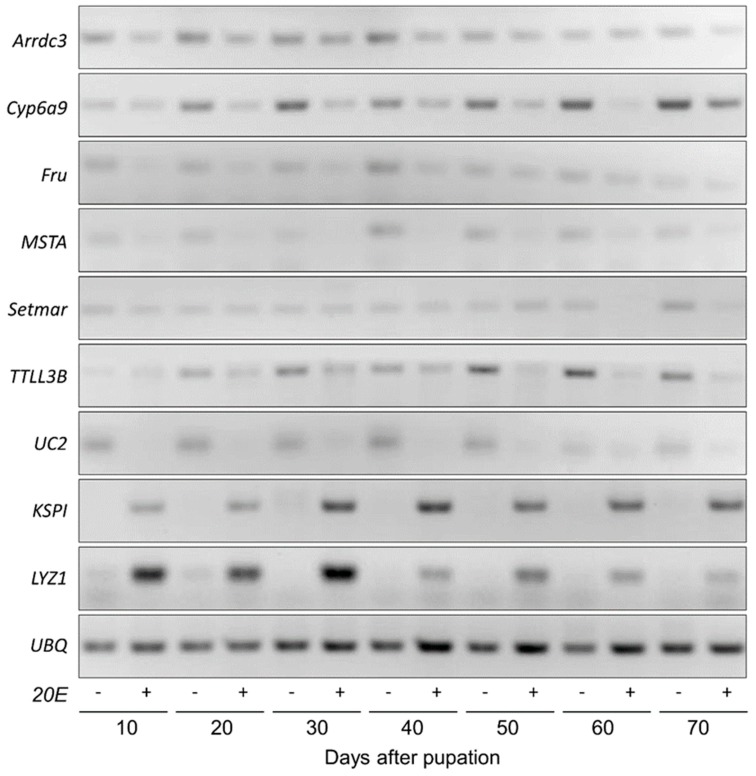
Relative gene expression levels between diapause-destined and non-diapause-destined *B. minax* pupae measured by semiquantitative PCR.

Subsequently, the semiquantitative PCR was conducted to further validate the association of these nine genes with diapause. The expression levels of 7 genes, *Cyp6a9*, *Fru*, *KSPI*, *LYZ1*, *MSTA*, *TTLL3B*, and *UC2* were consistent with the qRT-PCR data, displaying significant differences between diapause-destined and non-diapause-destined pupae. Nevertheless, *Arrdc3* and *setmar* only exhibited slight differences between diapause-destined and non-diapause-destined pupae (Figure 4). Taken together, *Cyp6a9*, *Fru*, *MSTA*, *TTLL3B*, and *UC2* are highly positively associated with diapause, while *KSPI* and *LYZ1* are highly negatively associated with diapause. The identities of these diapause-associated genes, except *UC2*, were confirmed by phylogenetic analyses (Figures S1–S6).

## 4. Discussion

According to our previous transcriptomic study, over 4000 genes were regulated throughout the pupal stage of *B. minax*, and several physiological pathways were deployed in diapause programming [17]. Despite the high sensitivity and accuracy in revealing the gene expression profile, the transcriptomic analysis still has errors. Therefore, the subsequent qRT-PCR validation is usually adopted [17,25,26]. In this study, 18 DEGs of top hits and displaying stable expression among biological replicates were manually selected as putative diapause-associated genes. The expression patterns of these 18 genes throughout the pupal stage were evaluated by qRT-PCR analysis, and 13 of them were consistent with that of previous transcriptomic study. Then, the newly-formed pupae were injected with 20E and solvent to acquire non-diapause-destined and diapause-destined pupae, respectively. Along with pupal development, the expression levels of positively diapause-associated genes ought to be increased with intensification of diapause in diapause-destined pupae, but not in non-diapause-destined pupae. In contrast, the negatively diapause-associated genes were expected to be activated in non-diapause-destined pupae but suppressed in diapause-destined pupae. Therefore, the qRT-PCR and semiquantitative PCR were performed to assess the differences in expression levels of remaining 13 genes between diapause-destined and non-diapause-destined pupae. The findings of these two methods were not exactly consistent with each other as *Arrdc3* and *setmar* displayed a significant difference in qRT-PCR analysis, but not in semiquantitative PCR analysis, probably due to the relatively higher sensitivity of qRT-PCR. Only seven genes which showed a significant difference in both analyses were deemed to be highly diapause-associated genes.

TTLL3B, a member of tubulin-tyrosine ligase (TTLL) family, catalyzes the polyglycylation of proteins, especially tubulin [29]. Polyglycylation is the long-chain glycines modification at a single or multiple glutamate sites on the C-terminal tail of protein, and the long-chain glycine is linked to the γ-carboxyl group of the glutamic acid residue via isopeptide bond. This modification consists of two steps, initiation and extend [30,31,32,33]. To date, glycylation has been found solely in axonemes of cilia and flagella [30,31,32,33,34,35], playing roles in stability of cell cilia and male sterility. However, little is known about its function in insects. To our knowledge, this is the first time that glycylation has been found associated with diapause, while its functions necessitate further investigation.

P450s are involved in the metabolism of exogenous substances such as insecticides and plant secondary metabolites, and also endogenous compounds like juvenile hormones, 20-hydroxyecdysone, ecdysteroids, and pheromones [36]. The CYP6a9 identified in this study belongs to P450 CYP6 family which is specific to insect [37]. Likewise, some members in P450 CYP6 family were also found to be associated with diapause of other insects, such as *Drosophila montana* [26]. It has been established that P450 genes are related with insecticide resistance, stress resistance, and immunity in organisms [38,39,40]. However, the roles *CYP6a9* plays in *B. minax* diapause remains unknown.

MSTA is a member of SET (suppressor of variegation, enhancer of zeste, trithorax) and MYND (myeloid-Nervy-DEAF1) domain-containing proteins family (SMYD) which are special protein lysine methyltransferases involved in methylation of histones and nonhistone targets [41,42,43]. The SET domain is an evolutionarily conserved catalytic unit for lysine methylation [44,45], and the MYND domain is a conserved zinc-binding domain primarily involved in protein–protein interaction [46]. SMYD family consists of six groups, SMYD1–5 in mammal [47], and arthropod-specific SMYDA [48]. SMYD proteins are related to epigenetic control, development, and cell proliferation through posttranslational modifications in histones and nonhistones. In insects, the studies on SMYD proteins are still limited and mainly focused on fruit flies. Knockdown of *Drosophila Smyd4* leads to abnormal eclosion, suggesting the vital role for *Smyd4* in development [49]. That may also be the case in diapausing *B. minax*.

*Fru* gene encodes a sex-specifically spliced transcription factor containing the zinc finger and BTB (Broad-complex, Tramtrack, and Bric-à-brac) domain [50,51,52]. It plays a crucial role in the courtship behavior and neuronal sex differentiation of male fruit flies [51,53]. For example, the female fruit flies expressing male-specific *Fru* in the nervous system showed the courtship behavior to other females, while the male flies expressing female-specific *Fru* developed into sterile males, lost the intrinsic properties of males, and exhibited female characteristics [54,55]. Knockdown of *Fru* gene in *Schistocerca gregaria* interfered mating and also affected fertility and fecundity of males [56]. Therefore, the *Fru* gene has been considered as a switch gene for courtship and thus contributing to the survival and reproduction of insects. However, the role *Fru* plays in insect diapause has not yet been studied. It is likely that certain unknown downstream genes other than courtship-related ones were activated by *Fru* in diapausing *B. minax*.

*KSPI* identified in this study is a serine protease inhibitor which modulates the bioactivity of target proteins through proteolytic cleavage. *KSPI* belongs to Kunitz-type protease inhibitor family, also known as bovine pancreatic trypsin inhibitor (BPTI)-like proteases [57]. As a classic representation of this family, BPTI has a broad inhibitory activity and mainly inhibits trypsin, chymotrypsin, and elastase-like serine protease [58]. The structures of all known Kunitz-type protease inhibitors of invertebrates are all similar to BPTI [59]. Serine protease inhibitors play important roles in the innate immunity of insects. It has been demonstrated that Kunitz-type protease inhibitors can be deployed to defense against microorganism invasion in insects [60,61]. Therefore, the downregulation of *KSPI* may suppress the immune response in diapausing *B. minax*, which is consistent with the findings that the immunity was attenuated in diapausing *Samia cynthia pryeri* pupae [62].

Lysozyme is a vital bacteriolytic enzyme in insect innate immune system, catalyzing the hydrolysis of 1,4-beta-linkages between N-acetylmuramic acid and N-acetylglucosamine (NAG) residues in the peptidoglycan of the bacterial cell wall [63]. The lysozymes fall into six different classes: c-type, g-type, i-type, bacterial, T4 phage-type, and plant lysozymes based on the source and mechanism [64]. The *LYZ1* found in this study belongs to c-type. Insect lysozymes are produced in fat body and released into hemolymph. The activity of lysozyme in hemolymph increases dramatically when insects are infected with pathogens [65,66]. Like *KSPI*, the *LYZ1* was downregulated during *B. minax* diapause as well, supporting the speculation that the pupal immunity was suppressed during diapause.

## 5. Conclusions

The univoltine *B. minax* enters obligatory diapause in pupal stage, during which a large number of genes are regulated. In this study, 5 genes, *TTLL3B*, *Cyp6a9*, *MSTA*, *Fru*, and *UC2*, and 2 genes, *KSPI* and *LYZ1*, were demonstrated to be positively and negatively associated with diapause, respectively. Nevertheless, there could be other genes that are involved in the *B. minax* diapause programming were not analyzed in the present study. Therefore, more effort can be exerted to explore the relevant genes. To elucidate the mechanisms underlying *B. minax* diapause, the functions of these diapause-associated genes ought to be further investigated in the future.

## Figures and Tables

**Table 1:** Information of selected putative diapause-associated genes.

Gene	ID	Accession No.	Annotation
*Arrdc3*	BmUnigene27028.co	GBEY01019016.1	Arrestin domain-containing protein 3
*Cyp6a9*	BmUnigene26209.co	GBEY01017833.1	Cytochrome P450 6a9
*Fru*	BmUnigene22870.co	GBEY01013664.1	Sex determination fruitless protein type C
*GEF*	BmUnigene27885.c5	GBEY01020590.1	Ras guanine nucleotide exchange factor P
*grk*	BmUnigene18469.co	GBEY01008416.1	Protein gurken
*MEGF10*	BmUnigene26874.co	GBEY01018777.1	Multiple epidermal growth factor-like domains protein 10
*MSTA*	BmUnigene27964.co	GBEY01020759.1	Protein msta
*MYBLI*	BmUnigene27469.co	GBEY01019748.1	Myb-like protein I
*Pcd*	BmUnigene23179.co	GBEY01014042.1	Pterin-4-alpha-carbinolamine dehydratase
*setmar*	BmUnigene15667.co	GBEY01005630.1	Histone-lysine N-methyltransferase SETMAR
*TTLL3B*	BmUnigene14579.co	GBEY01004593.1	Tubulin glycylase 3B
*UC1*	BmUnigene24070.co	GBEY01015150.1	Uncharacterized protein
*UC2*	BmUnigene30159.co	GBEY01022960.1	Uncharacterized protein
*ECE-2*	BmUnigene27012.co	GBEY01018994.1	Endothelin-converting enzyme 2
*KSPI*	BmUnigene25112.co	GBEY01016431.1	Kunitz-type serine protease inhibitor
*LYZ1*	BmUnigene21628.co	GBEY01012185.1	Lysozyme 1
*twk-7*	BmUnigene25271.co	GBEY01016631.1	TWiK family of potassium channels protein 7
*wbl*	BmUnigene21615.co	GBEY01012168.1	Protein windbeutel

## Supplementary Materials

The following are available online at https://www.mdpi.com/2075-4450/10/6/169/s1, Table S1: Primers used for qRT-PCR and Semiquantitative PCR, Figure S1: Phylogenetic analysis of the *Cyp6a9*, Figure S2: Phylogenetic analysis of the *Fru*, Figure S3: Phylogenetic analysis of the *KSPI*, Figure S4: Phylogenetic analysis of the *LYZ1*, Figure S5: Phylogenetic analysis of the *MSTA*, Figure S6: Phylogenetic analysis of the *TTLL3*.
