# Supplementary material for: Expression Analysis Reveals the Association of Several Genes with Pupal Diapause in Bactrocera minax (Diptera: Tephritidae)
Source: Insects. 2019 Jun 13;10(6):169. doi: 10.3390/insects10060169 (PMC6628110; doi:10.3390/insects10060169)
Supplement: Supplementary file 1 [file insects-10-00169-s001.zip › suppl/insects-505159-Table S1.docx]

**Table S1.** Primers used for qRT-PCR and semiquantitative PCR.

| **Gene Name** | **Primer Sequence (5’ to 3’)** | **Amplification Efficiency** | **Semi-Quantitative PCR** | |
| --- | --- | --- | --- | --- |
|  |  |  | **TM (°С)** | **Cycles** |
| *Arrdc3* | CGCTCTAGGGATGGCAAAAC  TCGACTACCAGCGAGAGTTC | 97.96% | 58 | 30 |
| *MEGF10* | TTGTCAAGCGCTATGTACGC  ACGCACATTTCAGGACCAAC | 104.42% |  |  |
| *TTLL3B* | ACACTGGATGAAGAAGGCGA  GATTACGGCAACCATCCCAC | 94.41% | 60 | 32 |
| *Cyp6a9* | ATCGGACGCGATCTCTGATT  ATGTCTCAAAGCCACCCAGA | 96.26% | 60 | 30 |
| *GEF* | CCGAAGCCACATACCAACAC  CGCCACGCCTATAGAAATCG | 93.92% | 58 | 28 |
| *Pcd* | ACCTCTTCTGAATGCTGGCT  CTGAGACCGCCAACATCATG | 101.63% |  |  |
| *grk* | ACCTTCAACCACACCTCCAA  ACCATCAACACTACTGGCGA | 98.18% |  |  |
| *MSTA* | ATGGCTGGCGGTATAAGTGA  TTCACTGCACACCCATTTCC | 96.54% | 58 | 30 |
| *Fru* | AGGTCGACAAGTTGCAGTTG  CCTCGCCCACATTTATCGTG | 91.61% | 58 | 30 |
| *UC1* | CTACAATCAGCCACAACCGG  AGCACTACCGTTCATCCACA | 95.82% |  |  |
| *UC2* | TTTGGAGCAACAACGAAGCA  CTCAGCAGACGACGCATTAC | 98.17% | 58 | 28 |
| *MYBLI* | TTTGGCCAAGGTTTCTTCCG  GGAACAAACCGCCATCAGTT | 96.74% |  |  |
| *setmar* | AAAACGGTCACCTGGCTCTA  AAGTTTCTGCCAAGCGAGTG | 103.14% |  |  |
| *ECE-2L* | CGAGCATTTGGAGCGTAACA  ATTCCACATAACGCGCTGTC | 102.71% |  |  |
| *twk-7L* | CATCTGCTTCGCTCTGATCG  TCTTCCCACAGCAGCAGTAA | 94.85% |  |  |
| *KSPI* | AGTTCAAATTCGGTGGCTGC  TGCGTTGGAATGTGATGCTT | 92.76% | 60 | 28 |
| *wbl* | TGTGTGGAGCTGGATGAACT  GAACCTCAGACCCAGCTCTT | 96.59% |  |  |
| *LYZ1* | CGGTCGTTTCTCCTACAACG  GAAACATGGATTAATGCTGGGC | 93.06% | 55 | 32 |
