# Supplementary figures and images for: Expression Analysis Reveals the Association of Several Genes with Pupal Diapause in Bactrocera minax (Diptera: Tephritidae)
Source: Insects. 2019 Jun 13;10(6):169. doi: 10.3390/insects10060169 (PMC6628110; doi:10.3390/insects10060169)

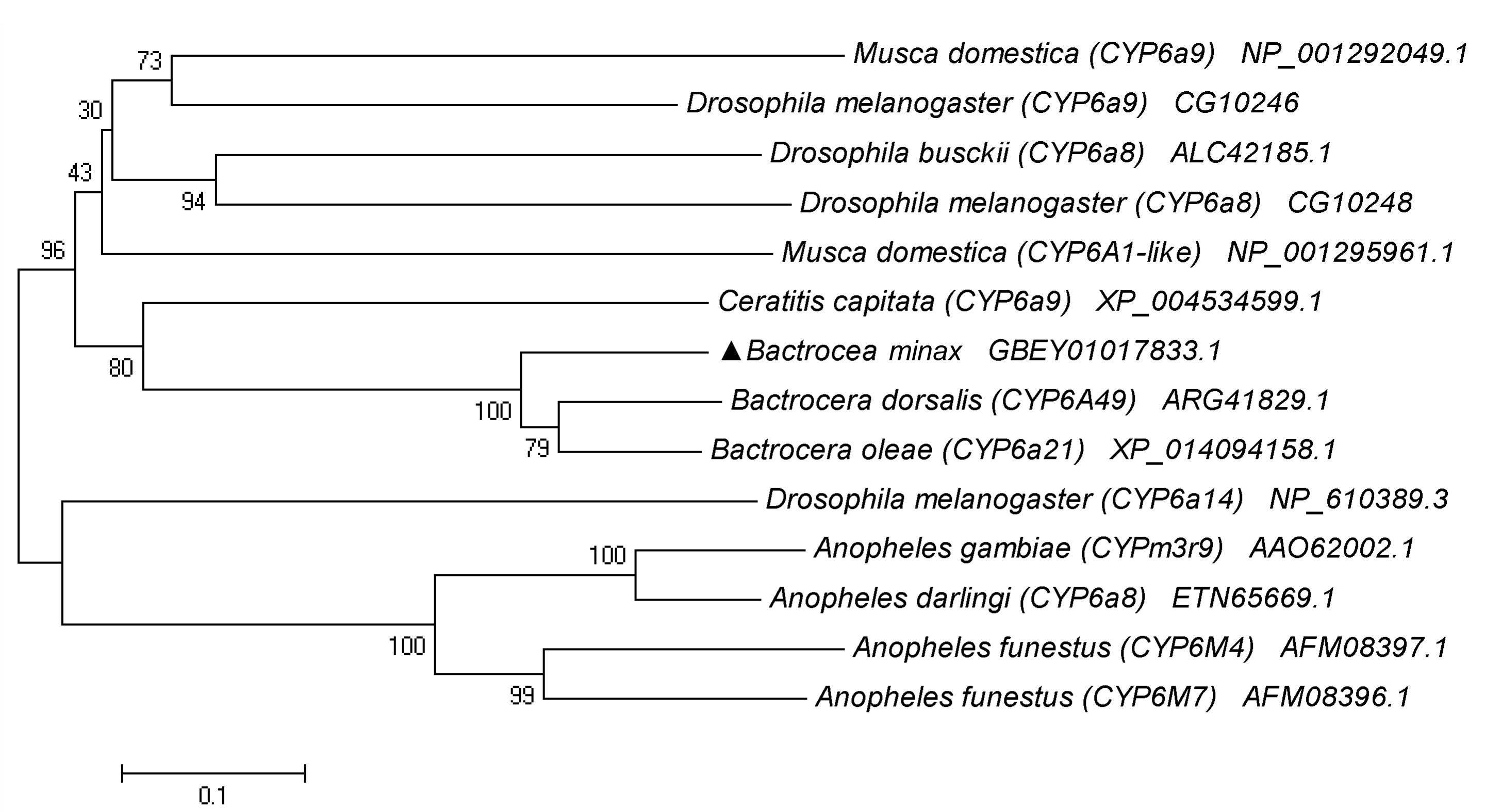

Supplement: Supplementary file 1 [file insects-10-00169-s001.zip › suppl/Figure S1.jpg]

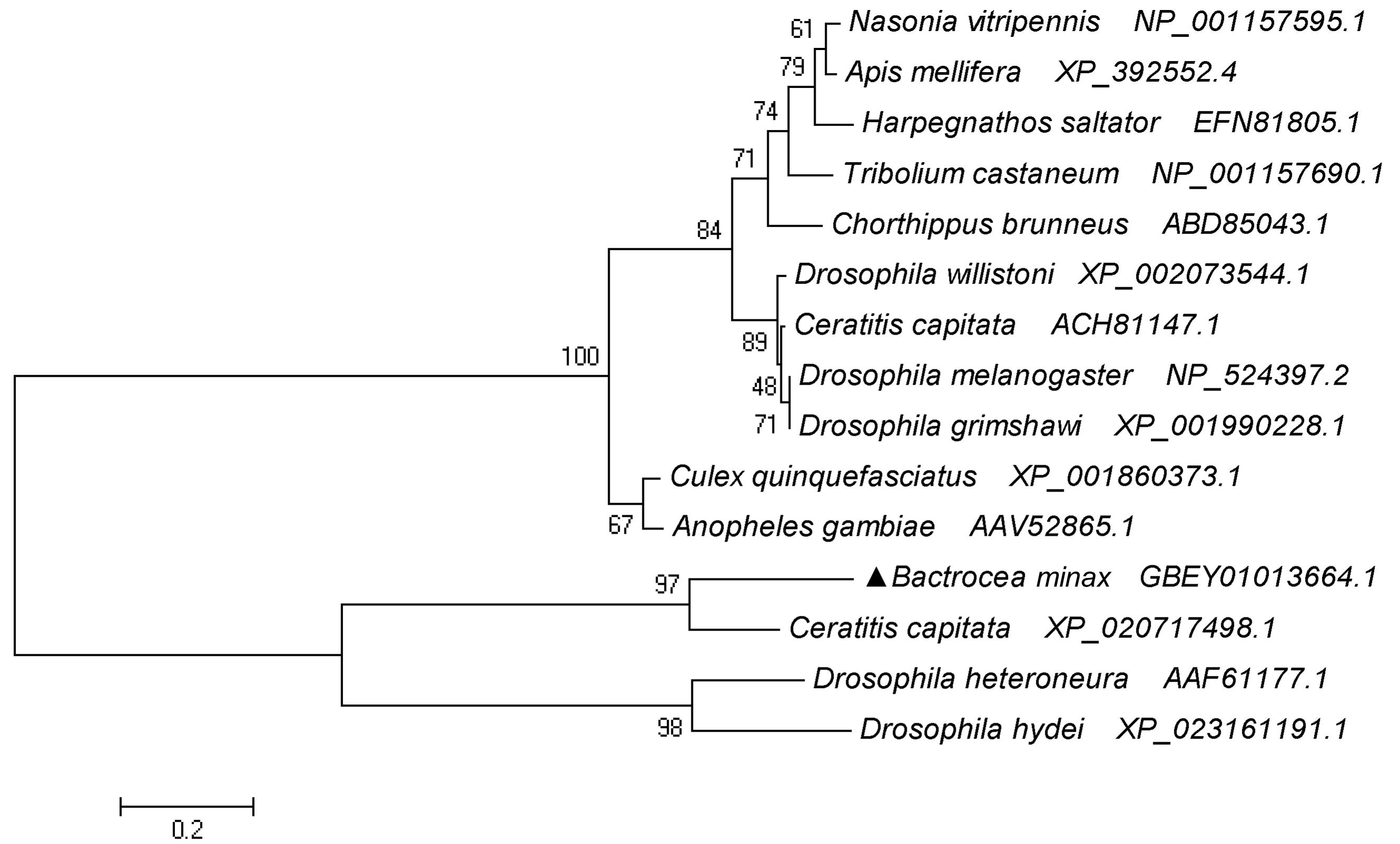

Supplement: Supplementary file 1 [file insects-10-00169-s001.zip › suppl/Figure S2.jpg]

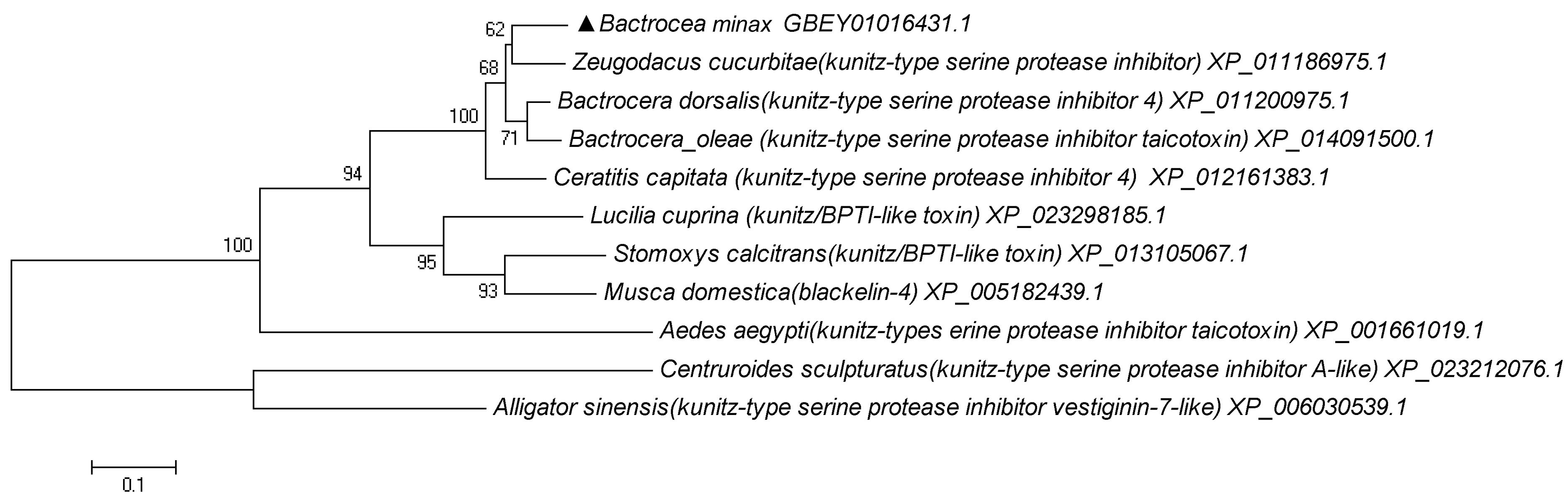

Supplement: Supplementary file 1 [file insects-10-00169-s001.zip › suppl/Figure S3.jpg]

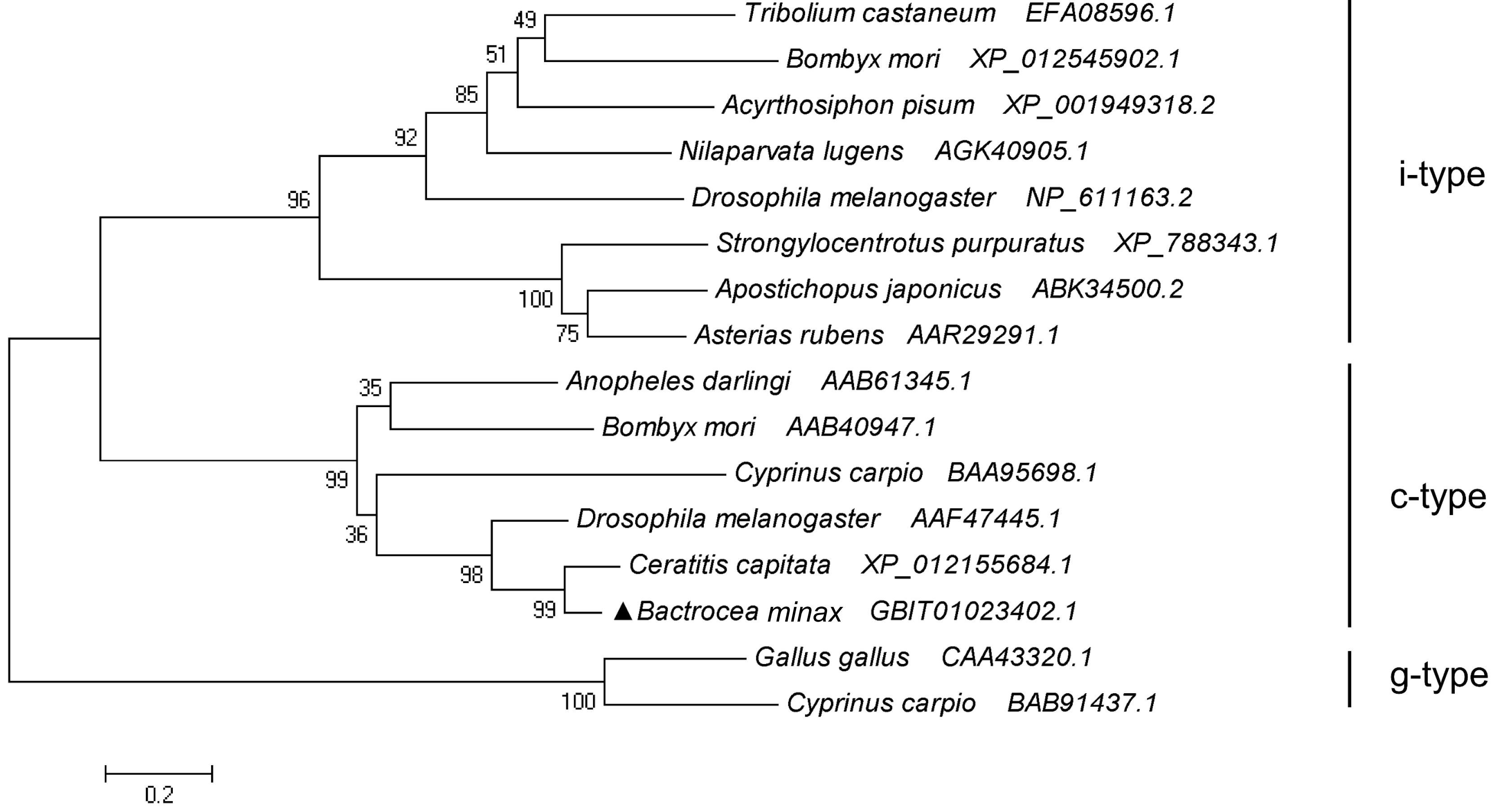

Supplement: Supplementary file 1 [file insects-10-00169-s001.zip › suppl/Figure S4.jpg]

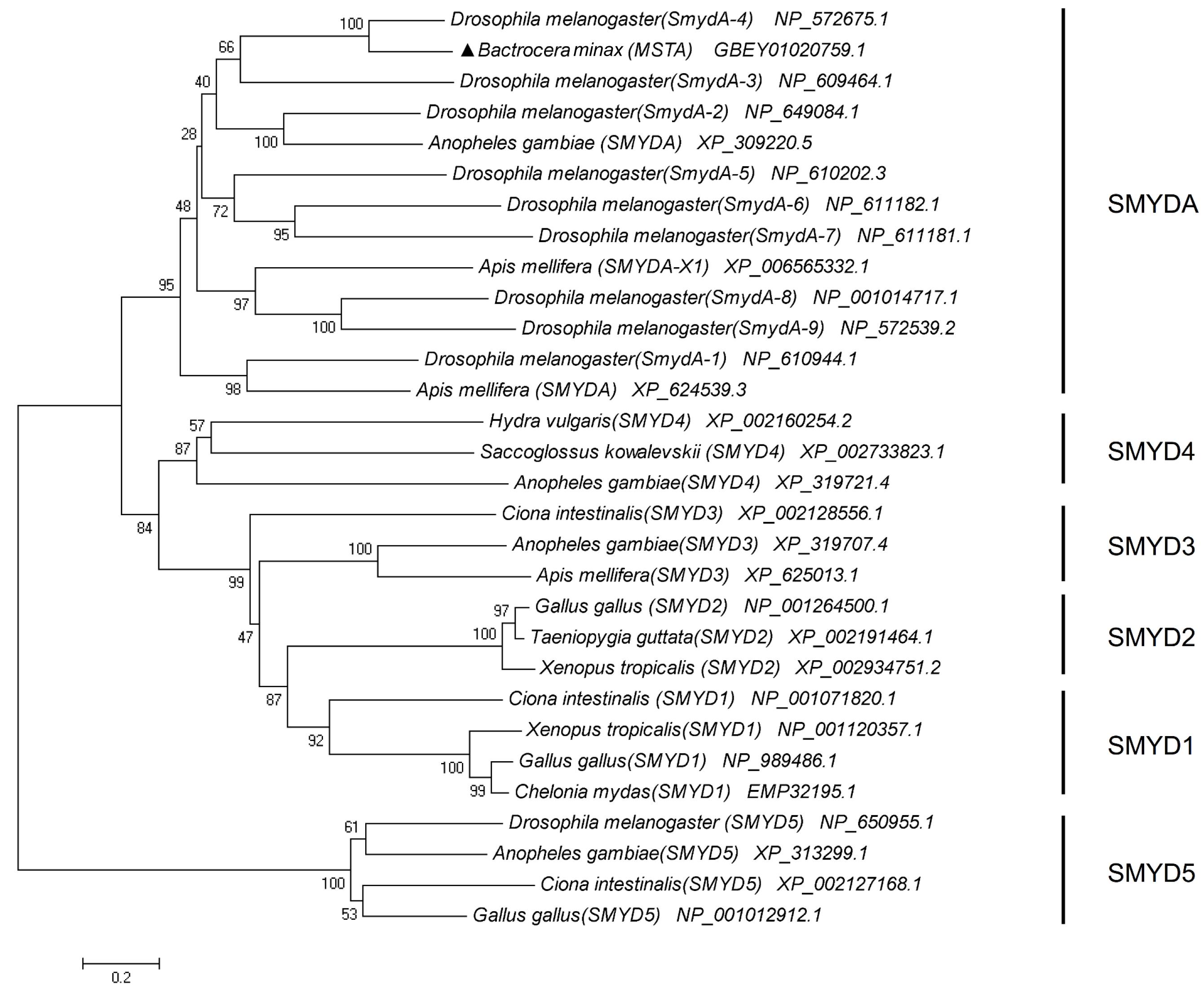

Supplement: Supplementary file 1 [file insects-10-00169-s001.zip › suppl/Figure S5.jpg]

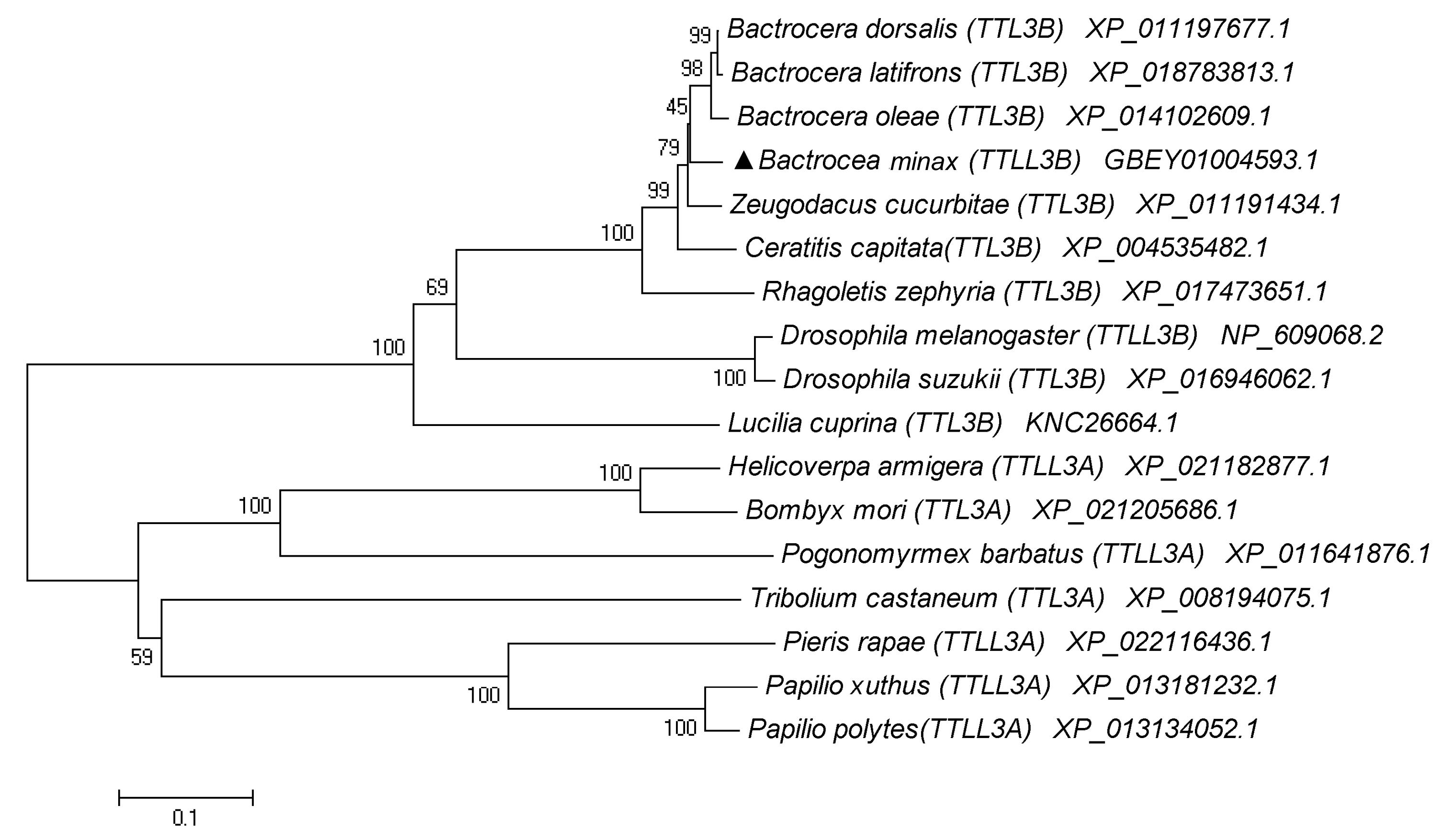

Supplement: Supplementary file 1 [file insects-10-00169-s001.zip › suppl/Figure S6.jpg]
